# Contrasting runoff trends between dry and wet parts of eastern Tibetan Plateau

**DOI:** 10.1038/s41598-017-15678-x

**Published:** 2017-11-13

**Authors:** Yuanyuan Wang, Yongqiang Zhang, Francis H. S. Chiew, Tim R. McVicar, Lu Zhang, Hongxia Li, Guanghua Qin

**Affiliations:** 1 Key Laboratory of Radiometric Calibration and Validation for Environmental Satellites, China Meteorological Administration (LRCVES/CMA), and the National Satellite Meteorological Center, China Meteorological Administration, Beijing, 100081 China; 2 CSIRO Land and Water, GPO Box 1700, Canberra, 2601 Australia; 3 ARC Centre of Excellence for Climate System Science & Climate Change Research Centre, University of New South Wales, Sydney, 2052 Australia; 4 State Key Laboratory of Hydraulics and Mountain River Engineering, Sichuan University, Chengdu, 610065 China

## Abstract

As the “Asian Water Tower”, the Tibetan Plateau (TP) provides water resources for more than 1.4 billion people, but suffers from climatic and environmental changes, followed by the changes in water balance components. We used state-of-the-art satellite-based products to estimate spatial and temporal variations and trends in annual precipitation, evapotranspiration and total water storage change across eastern TP, which were then used to reconstruct an annual runoff variability series for 2003–2014. The basin-scale reconstructed streamflow variability matched well with gauge observations for five large rivers. Annual runoff increased strongly in dry part because of increases in precipitation, but decreased in wet part because of decreases in precipitation, aggravated by noticeable increases in evapotranspiration in the north of wet part. Although precipitation primarily governed temporal-spatial pattern of runoff, total water storage change contributed greatly to runoff variation in regions with wide-spread permanent snow/ice or permafrost. Our study indicates that the contrasting runoff trends between the dry and wet parts of eastern TP requires a change in water security strategy, and attention should be paid to the negative water resources impacts detected for southwestern part which has undergone vast glacier retreat and decreasing precipitation.

## Introduction

As the highest plateau in the world, the TP plays an important role in the climate and hydrology of eastern and southern Asia^1–3^. Many large rivers, including Yangtze, Yellow, Brahmaputra, Indus, Ganges, Mekong and Salween rivers, originate from the TP and adjacent mountains, and these rivers sustain the lives of more than 1.4 billion people^4,5^. Over the past decades, the TP has been experiencing significant climatic and environmental changes, such as warming^6^, wetting^7^, drying^3^, dimming^3^, greening^8^, glacier retreat^9^, wind stilling^10,11^, permafrost degradation^12^, desertification^13^ and land use change^14^. These changes are characterized by pronounced regional disparities. Over the last 30–35 years southern and eastern TP which are impacted by South and East Asian monsoon have received decreased precipitation, whereas in contrast central and northern TP have experienced increased convective precipitation due to warming^3^. Glaciers in western TP, which are under the dominance of westerlies, show positive mass balance, while the glaciers in other parts of the TP show extensive shrinkage and negative mass balance^9^. Understanding the water resources availability response to these changes is fundamentally important for food security and regional sustainable development^2,14,15^.

Although hydrological models are very useful in understanding climatic and anthropogenic impacts on runoff (*Q*)^2,16,17^, it is not easy to model inter-annual variation of *Q* over the TP because of its unique climatic, topographic and hydrological features. Adapting hydrological models to simulate hydroclimate processes over the TP is challenging given that some hydrological processes (such as snow melt and refreeze, glacier retreat, permafrost degradation, soil moisture movement) are still poorly understood^18–21^. This is compounded by lack of streamflow gauges and meteorological stations (especially over high altitudes) to develop understanding of the key processes^22–24^, to elucidate hydroclimate changes over time, and to parameterize hydrological models.

We show that spatial and temporal variations of *Q* in eastern TP, where four large rivers (Yangtze, Yellow, Mekong, and Salween) originate from, can be reasonably estimated from individual water balance components derived solely from state-of-the-art satellite-based products (precipitation (*P*)^25^, actual evapotranspiration (*ET*)^26–29^ and change in total water storage (Δ*TWS*)^30^). We then analyze predominant features of the hydroclimate, and explain trends and variances in the water balance components, and uncertainties involved in the modelled *Q*.

## Results and Discussion

### Model validation

**Figure 1:**
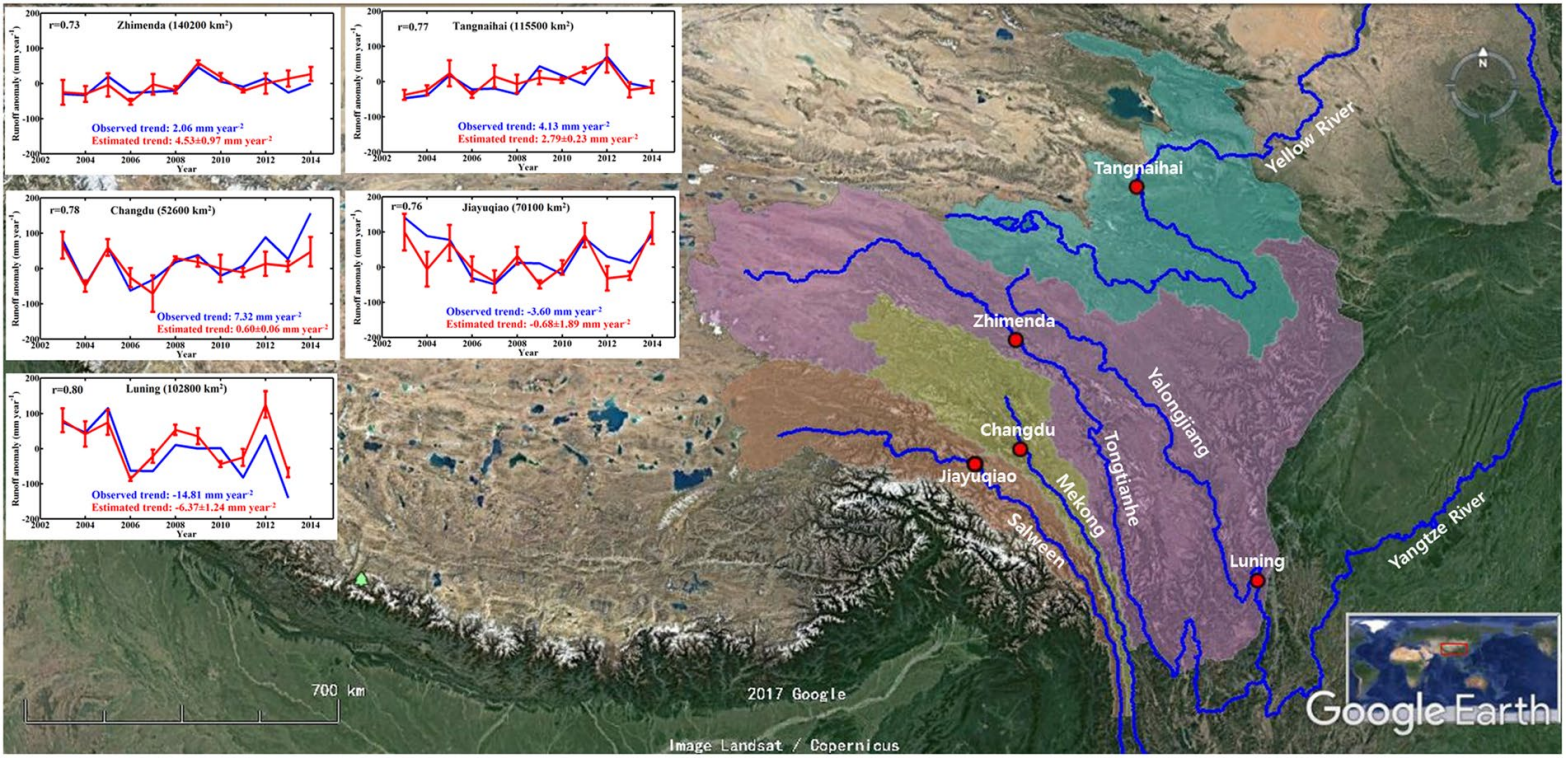
Comparison of observed and estimated *Q* anomaly time-series at the five gauges. Error bars are standard error generated from the four *ET* products. Luning basin is short of one year due to data missing. Blue lines are the river network. Red dots are the five streamflow gauges. Two gauges are for the Yangtze River (purple represents the Yangtze River basin) and one gauge is for each of the other three rivers (blue denotes the Yellow river basin, yellow represents the Mekong river basin, and orange the Salween river basin). Coloured area is the extent of eastern TP in this study. The map was generated using Google Earth (v 7.1.7.2606, https://www.google.com/earth/), Image Landsat/Copernicus.

We first compared the modelled (estimated from satellite products of *P*, *ET* and Δ*TWS*) and observed annual *Q* anomaly series from 2003 (Oct 2002 to Sep 2003) to 2014 (Oct 2013 to Sep 2014) and the linear trends in the 12 years of modelled and observed *Q* anomaly series at basin scale (Fig. 1). Mean values over 2004–2009 were used as reference for anomalies calculation, which is consistent with the mascon *TWS* product^30^. The modelled or satellite-data reconstructed annual *Q* anomaly series from the five basins agreed reasonably well with the observed annual *Q* anomaly series with linear correlation coefficients of 0.73 to 0.80. Furthermore, trends in the modelled and observed annual *Q* anomaly series were in the same directions though their magnitudes were noticeably different at three gauges. Trends in the observed *Q* at the five gauges varied within −14.8–+7.3 mm year^−2^, while trends in the reconstructed annual *Q* had a narrower range of −6.4–+4.6 mm year^−2^, with uncertainties of about 0.3–1.3 mm year^−2^.

### Explaining the variation in annual *Q*

**Figure 2:**
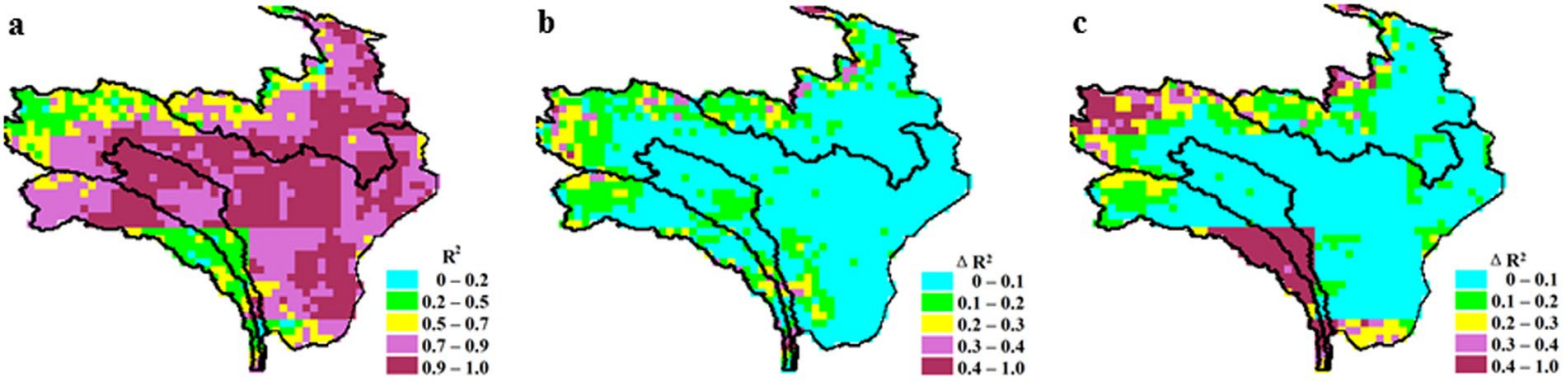
Contributions of water balance components to *Q* annual variation for eastern TP region shown in Fig. 1 for 2003–2014. (**a**), the coefficient of determination (R^2^) when only *P* is used to estimate *Q* variation. (**b**), additional R^2^ when *ET* together with *P* is used for estimating *Q* variation. (**c**), additional R^2^ when Δ*TWS* together with *P* is used for estimating *Q* variation. The maps were generated using ENVI (v 4.5, https://www.harris.com/solution/envi) © 2017 Harris Corporation.

Figure 2 shows that proportion of variance in annual *Q* can be explained by the three water balance components *P*, *ET* and Δ*TWS*. In practically the entire region, except for small parts in north and south-west, *P* explained more than 70% of variance in annual *Q*. *ET* had a relatively minor role in controlling the inter-annual variability of *Q*, whereas Δ*TWS* significantly enhanced the explained variance of annual *Q* in north and south-west. This is partly because *ET* was more stable than Δ*TWS*. Our results indicate that *ET* variance accounted for 6.1–7.6% of *P* variance, but this number rose to 13.1–16.4% for Δ*TWS* variance. Another reason was that Δ*TWS* reflected permafrost-induced hydrological changes in northern part^31–33^ and melting water amount from permanent snow/ice in southwestern part^34,35^.

### Spatial pattern of trends

**Figure 3:**
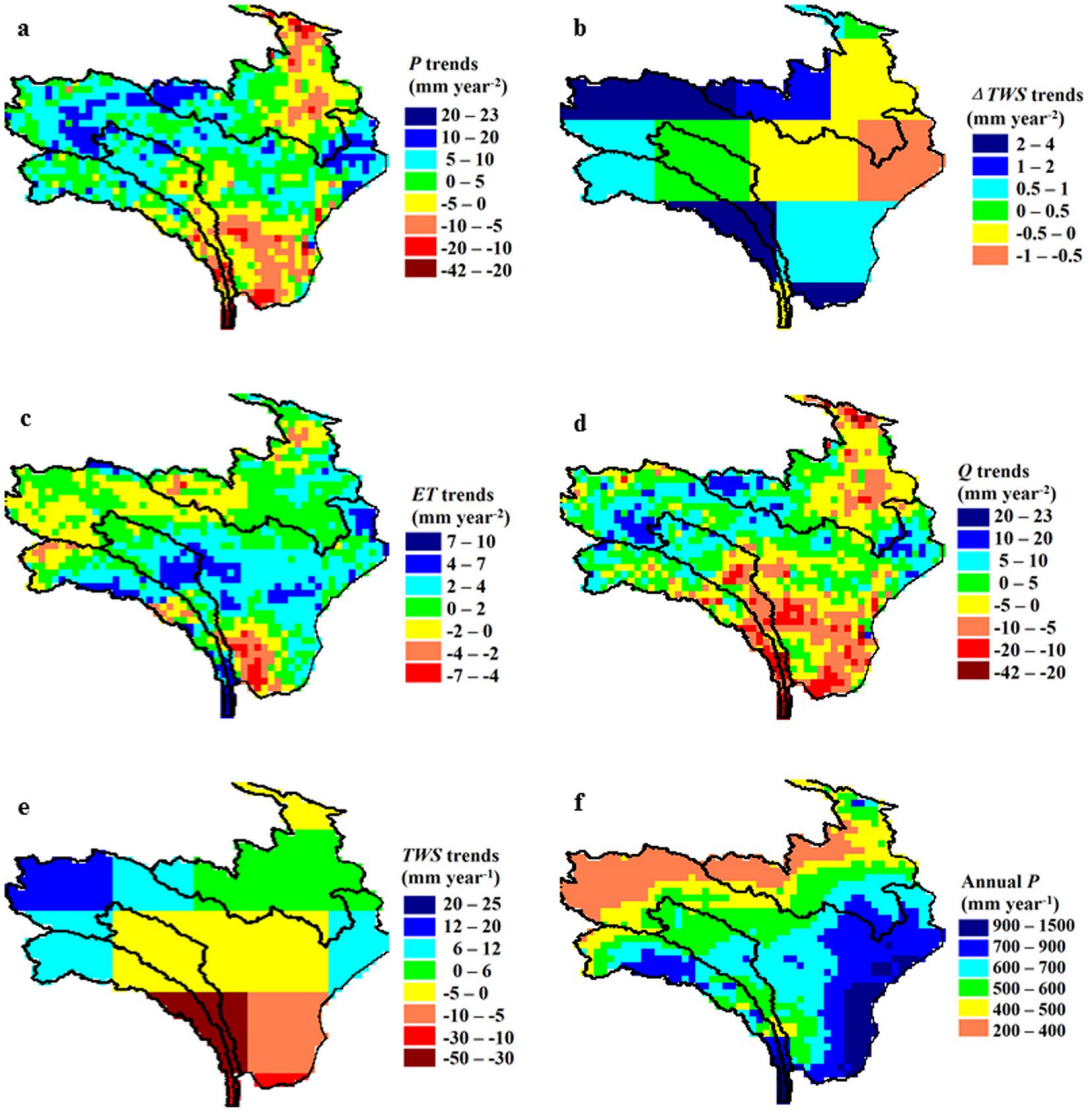
Spatial pattern of trends in *P*, *ET*, Δ*TWS*, *Q*, *TWS* and mean *P* values for eastern TP region shown in Fig. 1 for 2003–2014. The maps were generated using ENVI (v 4.5, https://www.harris.com/solution/envi) © 2017 Harris Corporation.

We further demonstrate spatial pattern of trends in *P*, *ET*, Δ*TWS* and *Q* over the recent 12 years (Fig. 3a–d). For interpretation purpose, we also show trends in *TWS* and distribution of multi-annual mean *P* (Fig. 3e,f). As expected, *Q* trends showed similar spatial pattern to *P* trends but had lower values because *Q* trends were moderated by slight changes or positive trends in *ET* and/or Δ*TWS*. *P* and *Q* increased clearly (+5–+23 mm year^−2^) in northwestern and eastern parts, decreased dramatically (−40–−10 mm year^−2^) in northern and southern parts. In middle and eastern parts, *ET* increased at +2–+7 mm year^−2^. For Δ*TWS*, increased trends were observed across most parts of the region, but the magnitudes were quite small (usually less than +2–+4 mm year^−2^). The *TWS* increased moderately (+6–+20 mm year^−1^) in northern part, but decreased substantially (−30–−50 mm year^−1^) in southwestern part, which is quite different from trends in Δ*TWS* because *TWS* is a state variable (with a unit of mm) while Δ*TWS* is a flux variable (with a unit of mm year^−1^).

Northern and southeastern parts, which are distinguished by dry and wet climates respectively (Fig. 3f), have undergone different changes that resulted in opposing *Q* trends. Increased trends in *Q* in the dry northern part were dominantly determined by increased trends in *P*. This is indicated by the aggregated trend in *Q* from regions with *P* less than 400 mm year^−1^ being +4.33 mm year^−2^, derived from trend in *P* being +6.40 mm year^−2^, compensated by trends in *ET* and Δ*TWS* being +0.17 mm year^−2^ and +1.90 mm year^−2^, respectively. In wet southeast, overall decreased trends in *Q* were similar to those in *P* trends, but in northern part of southeast, noticeable increased trends in *ET* (+4–+7 mm year^−2^) aggravated the decreased trends in *Q*. As a result, trend in aggregated *Q* from regions with *P* above 650 mm year^−1^ was −2.17 mm year^−2^, resultant from the trends in *P*, *ET* and Δ*TWS* being +0.58 mm year^−2^, +2.43 mm year^−2^, and +0.32 mm year^−2^, respectively.

### Contribution of Δ*TWS*

As expected, Δ*TWS* responds mainly to *P*, especially in dry regions (see Supplementary Information, Fig. S2). The reason that Δ*TWS* contributed greatly in explaining the inter-annual variation of *Q* in north and south-west is probably because Δ*TWS* can reflect cryosphere hydrological dynamics. In northern part, trends in both *TWS* and Δ*TWS* were clearly positive; respectively these indicate that total water storage increased and the rate of change increased too. To be more specific, we took Zhimenda basin located in north as an example. The whole 12 years were divided into two six-year periods. For the first six years (2003–2008), mean annual *P* and Δ*TWS* were 392 mm year^−1^ and +2.07 mm year^−1^, respectively. For the second six years (2009–2014), mean annual *P* and Δ*TWS* were 452 mm year^−1^ andn +7.33 mm year^−1^, respectively. The results clearly show that the total water stored in Zhimenda basin had a surplus since Δ*TWS* was positive over 2003–2014; and both *P* and Δ*TWS* increased over the two periods, but Δ*TWS* increased more quickly. This can be attributed to permafrost degradation^12^, which includes the process of overall permafrost thaw and/or active layer deepening^36^. From this recently activated store, more water could leak deep into subsurface, leading to a substantial increase of groundwater storage. This phenomena has been demonstrated in other studies^37,38^. Owing to a large proportion of *P* converted into groundwater storage, water available for runoff generation was reduced^39^. Trend in *Q* was largely determined by trend in *P*-Δ*TWS*.

In southwestern part, *TWS* trend was negative, contrasted by positive trend in Δ*TWS*, which means total water storage decreased but the rate has slowed or even reversed. This region has a high percentage of permanent snow/ice, and change in the mass of snow/ice can be reflected by Δ*TWS* ^9,32^ (see Supplementary Information, Fig. S3). Taking Jiayuqiao basin located in this area as an example, substantial decrease in *TWS* occurred over 2003–2006 when mean annual Δ*TWS* and *P* were −23.2 mm year^−1^ and 671 mm year^−1^, respectively. Over 2007–2014 mean annual Δ*TWS* and *P* were +0.31 mm year^−1^ and 677 mm year^−1^, respectively. It means that there existed previously a huge loss in snow/ice mass caused by warming (*P* hardly changed), and meltwater accounted for a large proportion of *Q*. Afterward, the remaining snow/ice didn’t change greatly (Δ*TWS* was approaching to 0), providing limited amount of meltwater. The fact that meltwater has stopped increasing after vast snow/ice shrinkage largely contributed to the decreased *Q* trend observed in Jiaoyuqiao basin.

### Contribution of *ET*

Globally *ET* is the second largest term in the terrestrial water balance after precipitation^40^. Compared to Δ*TWS* which is often assumed to be zero over a long period of time and a large region^41^, *ET* has more noticeable trends but with less intense fluctuations^26^. The small inter-annual variability of *ET* means that *ET* only provides a minor contribution in explaining *Q* variation, but its noticeable trends are indispensable in reconstructing *Q* trends. If the trends in *ET* were not considered, the *Q* trends would be severely over-estimated, especially for those basins with humid climates (Fig. 3). In other words, rapidly increased trend in *ET* in middle-south parts posed a threat to water resources availability, and this effect was more pronounced when *P* increased slightly or even decreased. Warming should be the main reason for the increasing trend in *ET* since *ET* of these wet regions are energy-limited^3,6^. Greening effect under the influence of CO_2_ fertilization could also play a role in enhancing *ET* ^26,42,43^.

### Uncertainty analysis

The water balance method was first validated at basin scale by comparing satellite estimated *Q* against gauge observations, and then applied to eastern TP for temporal-spatial analysis. An uncertainty may arise in this extrapolation process because the drainage areas upstream of the five gauges don’t fully cover the entire eastern TP. However, such uncertainty should be small because the five validated basins account for 59% of the research domain.

As *P* is the primary factor governing long-term *Q* and its variability, it is very important that our *P* data is as accurate as it can be. There was a good agreement in trends and Coefficients of Variation (CV) between Tropical Rainfall Measuring Mission satellite (TRMM) *P* data and local observations at 87 gauges located within the research domain (see Supplementary Information, Fig. S5a and S5c). With respect to inter-annual variations in *P*, observations show strong correlations with TRMM *P* data for most gauges (see Supplementary Information, Fig. S5b). Moreover, inter-annual variation in observed *Q* from the five gauges can be best explained with the TRMM *P* data, compared to those obtained from west01^44^, MSWEP^45^, nearest interpolation results of the monthly gauge-observed *P*, and thin-plate regression interpolation^46^ results using TRMM as a covariate (see Supplementary Information, Table S4). Therefore, we directly used raw TRMM *P* data without further processing.

**Figure 4:**
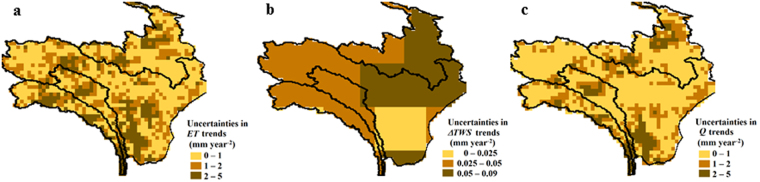
Uncertainties in *ET* trends, Δ*TWS* trends, and *Q* trends for eastern TP region shown in Fig. 1. (**a**), uncertainties in *ET* trends by considering the standard deviations of four *ET* products. (**b**), uncertainties in Δ*TWS* trends by considering the inversion error of mascon *TWS*. (**c**), uncertainties in reconstructed *Q* trends. Please note that uncertainties in Δ*TWS* trends are much smaller than the uncertainties in *ET* trends or in *Q* trends. *Q* trends are less uncertain than *ET* trends because *Q* trends are dominated by *P* trends. The maps were generated using ENVI (v 4.5, https://www.harris.com/solution/envi) © 2017 Harris Corporation.

GRACE mascon *TWS* data has a spatial sampling of 0.5 degree, but the native resolution of a single mascon are 3 degrees in both latitude and longitude, which explains why *TWS* or Δ*TWS* shows abrupt boundaries (Figs 2c, 3b,e and 4b). The course resolution can lead to high leakage errors for small catchments because surrounding signals are mistaken as the information from the catchment itself. It can be observed from Fig. 1 that the most inaccurate *Q* trend estimation occurred at the smallest catchment Changdu (52,600 km^2^) and its surrounding is rich in glaciers. Applying gain factors is a way to decrease leakage error^47^. However, no improvements in *Q* trend estimates were found after Δ*TWS* data were up-scaled to 0.5 degree using gain factors (see Supplementary Information, Fig. S4a). Moreover, the gain factors, which were derived from Community Land Model (CLM) and should be close to 1, show unreasonable values for some grids (see Supplementary Information, Fig. S4b). Therefore, these optional gain factors were not applied here. In addition to leakage error, another source of uncertainty is the inversion error of mascon *TWS* data. We found this error accounted for 3–20% of annual amplitude in *TWS*, resulting in an uncertainty of less than 0.09 mm year^−2^ in Δ*TWS* trends (see Fig. 4b), which negligibly affected the reconstructed *Q* trends.

Uncertainties in *ET* trends are usually less than 2 mm year^−2^, but for many grids of our study region, the values could be as high as 3–5 mm year^−2^. Trends in *ET* are much more uncertain than trends in Δ*TWS*, suggesting tremendous differences among the four *ET* products. The disparities could be caused by two factors: imperfectness or over-simplification of the *ET* model and low quality of input data (i.e., satellite data over eastern TP is usually of low quality because of cloud contamination and/or complicated terrain). The uncertainty in input data can be propagated into the *ET* estimates, leading to large inconsistencies among the models that are based on different concepts (see Supplementary Information, Fig. S1).

There was a strong resemblance in spatial pattern between uncertainties in *ET* trends and uncertainties in *Q* trends, indicating that the former largely influenced the latter. However, the latter were generally lower since *Q* trends were mainly controlled by *P* trends and significant *P* trends can supress the effect of uncertainties in *ET* trends. It can be seen from the Fig. 4c that *Q* trends had low uncertainties in dry northwestern and eastern regions and high uncertainties in the regions with annual mean *P* of 400–600 mm year^−1^, indicating that *Q* trends were more uncertain in moderate climate than in extreme (wet or dry) climate.

### Summary

This study focused on spatial representation of *Q* trends and its drivers over eastern TP solely using satellite-based products. Results indicated that *P* was the most important factor in determining *Q* trends and explained a major part of annual variation in *Q*. Δ*TWS* variations played an important role in regions undergoing distinct cryosphere dynamics, and *ET* trends were indispensable in determining *Q* trends in wet regions. Northern dry regions showed increased *Q* trends, whereas wet southern regions showed decreased *Q* trends, demanding for appropriate adjustments to future water resources management. This study also indicated that permafrost-induced hydrological change and glaciers melting are of great importance for *Q* modelling over the TP, suggesting that there still exists significant room for improving the current hydrological models.

## Methods

### Water balance equation

The annual water balance at each grid cell can be described as:

1 $$Q=P-ET-{\rm{\Delta }}TWS$$

where *Q* is annual runoff, *P* is annual precipitation, *ET* is annual actual evapotranspiration, and Δ*TWS* is change in Total Water Storage over the year. *TWS* is vertically integrated water stored, including groundwater, soil moisture, surface water, snow and ice, vegetation water, etc. The water year is defined as October to September to minimize effects of snowfall and glaciers on yearly water balances^48^. All variables have units of mm year^−1^. The baseline averages over 2004 to 2009 were used to calculate anomalies of water balance components.

### Data

All the above data were obtained from satellite-based products. Monthly precipitation data were sourced from the TRMM (3B43) precipitation product from Mirador at the NASA Goddard Earth Sciences Data and Information Services Center (GES DISC)^25^, which estimates 0.25^o^ gridded precipitation by merging 3B-42 and gauged precipitation data. Monthly *ET* data from Jan 2002 to Sep 2014 were obtained from four state-of-the-art diagnostic *ET* products: PML^26^, GLEAM^27^, MOD16^28^, *P*-LSH^29^. The median of *ET* anomalies from the four *ET* products were used for the analyses. The anomaly, rather than the actual value, was used to overcome systematic bias in the different *ET* products (see Supplementary Information Table S3 and Fig. S1). Monthly streamflow data for the five basins from Jan 2002 to Sep 2014, recorded by the Tibetan and Qinghai Hydrological Bureaus, were obtained from the hydrological annuals compiled by the Chinese Ministry of Water Resources. Monthly *TWS* data from Apr 2002 to Mar 2016 came from the Jet Propulsion Laboratory (JPL)-RL05M Mascon (mass concentration) Version 2. The mascon data is essentially another form of gravity field basis functions and is superior to the standard spherical harmonic approach due to implementation of prior geophysical constraints and less dependence on gain factors^30^. Δ*TWS* is calculated as *TWS* at the end of a water year minus *TWS* at the beginning of a water year.

### Statistical analysis

12 water years of data, from 2003 (Oct 2002 to Sep 2003) to 2014 (Oct 2013 to Sep 2014), were used for the analysis. First, basin-scale modelled annual *Q* anomaly series were assessed against the *Q* observations. Weighting averaging was used to obtain basin-average *P*, *ET*, and Δ*TWS* values, in which a grid’s weight is the ratio of this grid’s area inside the basin boundary (please note that the basin is the drainage area upstream of each gauge). Then, the annual series of *Q* anomaly were calculated for all 0.25^o^ grids across the region using Equation (1). The percentage of variance in *Q* that can be explained by *P*, by both *P* and *ET*, and by both *P* and Δ*TWS* was estimated as the coefficient of determination (R^2^) of the linear regression of *Q* versus *P*, *Q* versus *P* and *ET*, and *Q* versus *P* and Δ*TWS*, respectively. The trends in *Q*, *P*, *ET* and Δ*TWS* over the 12 years were calculated using the non-parametric Mann-Kendall test^49^. The uncertainties in *ET* trends were derived from the differences between trends in *ET* + *sd*_*ET*_ and trends in *ET*−*sd*_*ET*_. The uncertainty in *ET* (denoted as *sd*_*ET*_) is the standard deviations of the four *ET* anomalies. The uncertainties in Δ*TWS* trends were derived from the differences between trends in Δ*TWS* + *sd*Δ_*TWS*_ and trends in Δ*TWS*−*sd*Δ_*TWS*_. The uncertainty in Δ*TWS* (denoted as *sd*Δ_*TWS*_) is the squared mean of uncertainties of *TWS* ^30^ at the beginning and the end of a water year.

## Electronic supplementary material

Supplementary Information
