# Supplementary material for: Contrasting runoff trends between dry and wet parts of eastern Tibetan Plateau
Source: Sci Rep. 2017 Nov 13;7:15458. doi: 10.1038/s41598-017-15678-x (PMC5684371; doi:10.1038/s41598-017-15678-x)
Supplement: Supplementary file 1 — Supplementary Information [file 41598_2017_15678_MOESM1_ESM.pdf]

Supplementary information

Contrasting runoff trends between dry and wet parts of eastern Tibetan Plateau

Yuanyuan Wang[1,2], Yongqiang Zhang[2], Francis H.S. Chiew[2], Tim R. McVicar[2,3], Lu Zhang[2], Hongxia Li[4], Guanghua Qin[4]

[1]*Key Laboratory of Radiometric Calibration and Validation for Environmental Satellites, China Meteorological Administration (LRCVES/CMA), and the National Satellite Meteorological Center, China Meteorological Administration, Beijing 100081, China*

[2]*CSIRO Land and Water, GPO Box 1700, Canberra 2601, Australia*

[3]*ARC Centre of Excellence for Climate System Science & Climate Change Research Centre, University of New South Wales, Sydney 2052, Australia*

[4]*State Key Laboratory of Hydraulics and Mountain River Engineering, Sichuan University, Chengdu 610065, China*

This file includes five sections

1. Characteristics of the five basins
2. Evapotranspiration products introduction
3. ΔTWS response to precipitation and snow cover change
4. Application of gain factors to ΔTWS
5. TRMM precipitation validation

1 Characteristics of the five basins

Tangnaihai basin contributes to Yellow river. Luning and Zhimenda basins are the upstream of Yangtze River, but correspond to Yalongjiang and Tongtianhe streams respectively. Jiayuqiao basin is upstream of Salween River. Changdu basin is the upstream of Mekong River. Table S1 and Table S2 summarized basin characteristics. Changdu basin is the smallest, while Zhimenda basin is the largest. Jiayuqiao basin shows the highest percentage of permanent snow/ice cover, followed by Zhimenda basin. The three basins (Tangnaihai, Changdu, and Zhimenda) located in the Three Rivers Source Region are cold and dry. Particularly, Zhimenda is the coldest and driest, with the highest percentage of barren land. Compare to these, the other two basins are relatively wetter and warmer. All basins have mean elevations larger than 4000 meter above sea level. Zhimenda and Jiayuqiao basins have the highest elevation. Although Luning basin is not very high, it has the largest standard deviation of elevation, meaning it has the most complex topographic features. Another prominent feature of Luning basin is its high forest coverage (23.57%). In terms of runoff coefficient (mean annual runoff (Q) divided by mean annual precipitation (P)), Tangnaihai and Zhimenda are dry, with runoff coefficient less than 0.3, while the other three are wet, with runoff coefficients exceeding 0.45.

Table S1. Climatic and topographic features for each basin. Elevation statistics were calculated from GTOPO30; Climate data were obtained from Worldclim dataset[1].

Gauge name	Basin	Stream	Area (10^4 km^2)	Mean elevation (m)	Std of elevation (m)	Mean Annual *P* (mm $year^{-1}$)	Mean Annual *T* (° C)	Runoff coefficient
Tangnaihai	Yellow	Yellow	11.55	4122	410	540	-1.79	0.29
Changdu	Mekong	Mekong	5.26	4549	364	586	-0.96	0.48
Zhimenda	Yangtze	Tongtianhe	14.02	4753	275	404	-4.52	0.27
Jiayuqiao	Salween	Salween	7.01	4770	353	690	-1.86	0.54
Luning	Yangtze	Yalongjiang	10.28	4077	602	752	1.95	0.53

Table S2. Land cover characteristics for each basin. Percentage (%) of each land cover type for each basin was calculated from MCD12 product in 2006.

Gauge name	Water body	Forest	Grassland & shrub	Farmland	Residential	Permanent Snow/ice	Barren land
Tangnaihai	0.99	0.09	97.57	1.12	0.04	0.09	0.09
Changdu	0	1.18	97.13	0.87	0.04	0.62	0.16
Zhimenda	0.35	0.09	89.47	0.13	0.00	0.99	8.96
Jiayuqiao	0.27	1.47	93.61	0.86	0.01	3.04	0.75
Luning	0.04	23.57	73.50	2.42	0.02	0.31	0.14

2 Evapotranspiration products introduction

Evapotranspiration (*ET*) models are usually very complicated, requiring remote sensed data, meteorological data, and other ancillary information (such as land cover type, land surface roughness, DEM etc.)[2]. It is expected that uncertainties could be large from a single *ET* product because of errors in input data, imperfect modeling of physical process, error propagation and failure of achieving global optimum solution in parameterization. To reduce the uncertainties and bias, we obtained four state-of-the-art diagnostic *ET* products (PML[3], GLEAM[4], MOD16[5], *P*-LSH[6]) that are heavily driven by satellite data together with climate data, and used the model ensemble to calculate median anomaly. Note that calculating the anomaly excludes

any systematic bias for a particular product which could be large due to an absence of the water balance constraint. Summary information of the four *ET* products is listed in Table S3. Among them, GLEAM is based on Priestly-Taylor (PT) approach, and the other three are based on Penman-Monteith (PM) approach. Their input data and the modeling process are largely different, so it can be assumed that these products are independent and an ensemble of these products can increase the accuracy of *ET* anomaly estimates.

Table S3. Summary of *ET* products used in this study.

Product name	Length / Temporal Resolution	Spatial resoluti-on (d)	Validation accuracy (according to the author)	Brief description of the method used
PML	1981-2011/(monthly)	0.5	R^2=0.87 for annual *ET* (643 watersheds) R^2=0.77 for monthly *ET* (95 FLUXNET towers)	A modified PM equation (PML model). For each grid cell, the PML model is constrained by the classic Budyko framework, the hydroclimatic model that derives mean annual evaporation rates.
GLEAM	1980-2014/(daily)	0.25	R=0.83-0.86 for monthly *ET* (163 FLUXNET towers)	Priestly-Taylor approach by using the dataset that are based on reanalysis net radiation and air temperature, satellite and gauged-based precipitation, VOD (Vegetation Optical Depth) and snow water equivalents.
MOD16	2000-2014/(monthly)	0.05	R=0.86 for average daily *ET* (46 flux tower sites)	PM equation by using MODIS product (fPAR, LAI, albedo, NDVI, land cover) and spatially interpolating GMAO (Global Modelling and Assimilation Office) reanalysis data.
P-LSH	2002-2013/(monthly)	0.083	R^2=0.81 for annual *ET* (284 watershed)	Process-based Land Surface Evapotranspiration/Heat Fluxes algorithm. A modified PM approach with AVHRR NDVI-determined canopy conductance is used for calculate land *ET* and a Priestly-Taylor approach to calculate open water evaporation.

To show spatial pattern of the four *ET* products in our study region, we mapped mean annual *ET* for each product over 2004–2009 (i.e., the reference period for anomaly calculation). All the products generally show wet-to-dry gradient from

southeast to northwest, but differ greatly in the absolute values and spatial details (Fig. S1). For example, regional mean value of P-LSH product is almost twice that of PML product; GLEAM and MODIS have similar regional mean values but show very different spatial pattern. Nonetheless, these differences are expected, justifying the necessity of applying the median anomaly analysis for the four *ET* products.

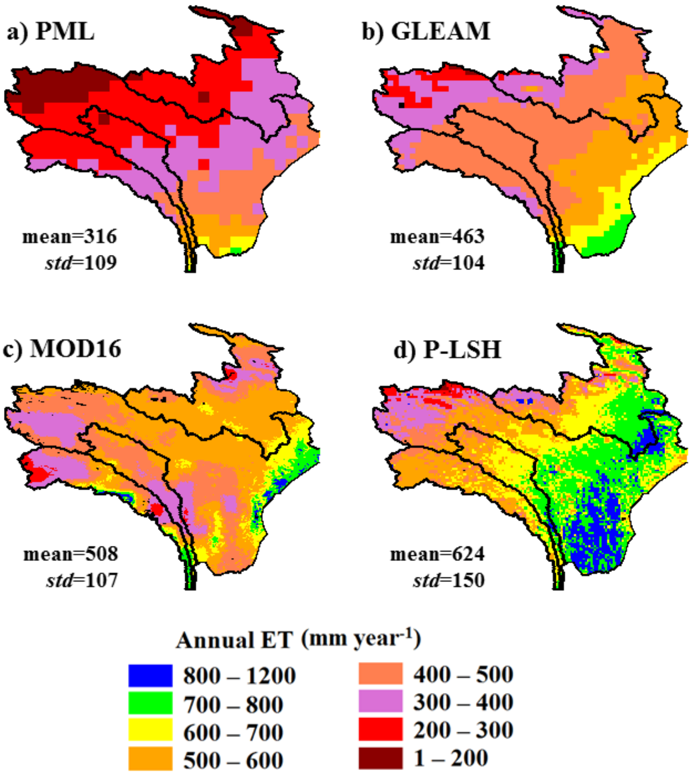

Fig S1. Multi-annual mean *ET* (2004–2009) of the study region derived from the four products. Regional mean and standard deviation (*std*) values are also provided for interpretation. The map was generated using ENVI (v 4.5, https://www.harris.com/solution/envi) © 2017 Harris Corporation.

3 ΔTWS response to precipitation and snow cover change

In most regions, ΔTWS and P show strong correlations, especially in northern

part, indicating that P drives ΔTWS. For other regions featured with wetter climate, the correlations are weak (Fig. S2). This is consistent with previous findings that ΔTWS of catchments in dry climate zones are more sensitive to P than catchments in humid climates[7].

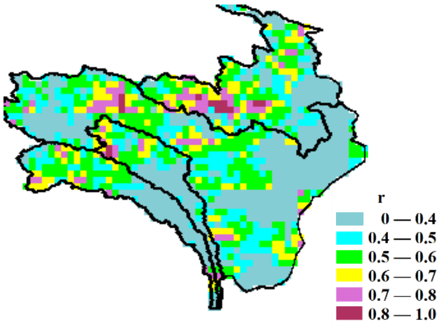

Figure S2. Map of correlation coefficient between annual ΔTWS and P over 2003-2014 for the study site. The map was generated using ENVI (v 4.5, https://www.harris.com/solution/envi) © 2017 Harris Corporation.

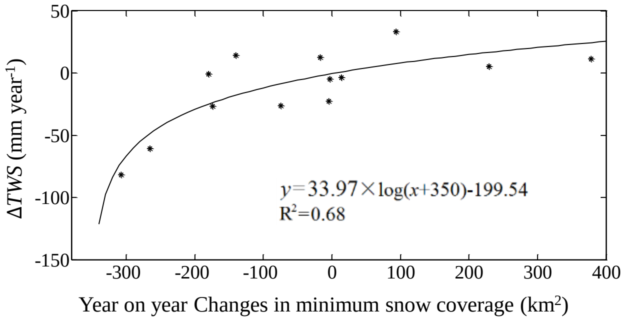

Figure S3. Scatterplot of ΔTWS and year-on-year changes in permanent snow coverage (derived from MOD10 product) for Jiayuqiao basin over 2003–2014.

Mass change of permanent snow/ice could be a driving force for ΔTWS in the south-western region of the study site. Due to a lack of annual ice/snow mass

information, we used annual minimum snow coverage as a proxy. The MODIS snow cover product data (MOD10) at 0.05 degree spatial resolution were processed to delineate annual minimum snow cover assuming snow that doesn't melt in summer is the permanent snow/ice[8]. We further explored the relationship between ΔTWS and the year-on-year changes in permanent snow coverage for Jiayuqiao basin of Salween River located in south-western region (Fig. S3). ΔTWS is strongly non-linear correlated to permanent snow/ice cover change, and becomes more sensitive when the area of permanent snow/ice coverage is reduced by more than 200 km^2 compared to the previous summer value. This reflects the non-linear relationship between snow/ice area change and mass change[9].

4 Application of gain factors to ΔTWS

Due to the destriping and smoothing effect caused by filters, gain factor usually need to be applied to the GRACE solutions over land. However, as far as mascon (mass concentration blocks) data is concerned, it is not a mandate to apply gain factors[10] since geophysical constrains are implemented in calculating mascon fields and the gain factors are very close to 1. Nonetheless, we still compared two sets of Q results obtained with and without gain factors. No substantial difference was observed for the five basins, confirming that Q estimation performance cannot be improved by applying gain factors (Fig. S4a). We further investigated the spatial pattern of gain factors with spatial resolution of 0.5 degrees (Fig. S4b), and found that for most of our study site they are close to 1 (i.e., the expected value), yet in the northwest, both extremely small (even negative) and large values exist. This is unreasonable and is

likely caused by the inadequacy of the Community Land Model (CLM) in modelling hydrological process over northwest which has widespread permafrost and where two big lakes Lake Zhaling and Lake Eling are located with the areas 528 km^2 and 628 km^2, respectively[11]. Although these abnormal gain factors don't affect Q results aggregated at basin scale, they could have negative impacts at grid level, so we chose to use raw ΔTWS data despite of its coarse resolution (3 degrees).

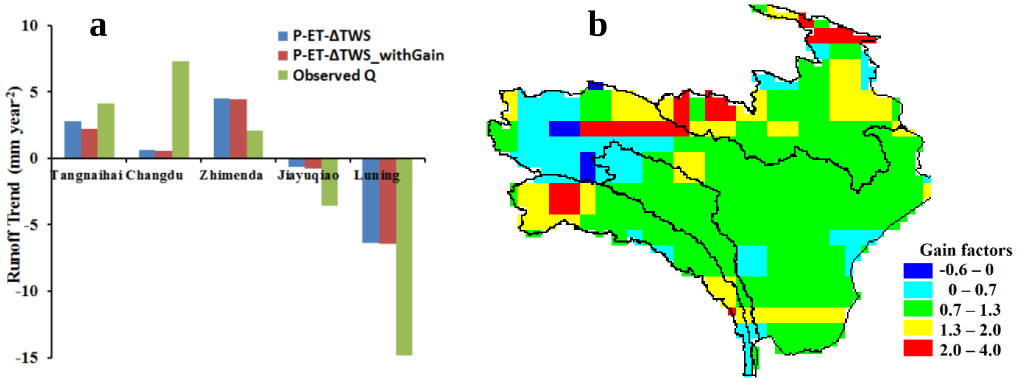

Figure S4. (a) Q trend comparison for the five basins. Applying gain factors doesn't obviously impact the trend in the reconstructed Q. **(b) Gain factors of mascon field for our study site.** The map was generated using ENVI (v 4.5, https://www.harris.com/solution/envi) © 2017 Harris Corporation.

5 TRMM precipitation validation

We first evaluated TRMM P data against local observations at 87 meteorological stations located within the modelling domain. There was a high consistency in the observed and TRMM P trends (Fig. S5a). For most stations, P observations were strongly correlated TRMM data (r > 0.75), indicating a reasonable match in inter-annual variations (Fig. S5b). The TRMM data also captured dispersion features

of probability distribution in *P* observations, which is measured by Coefficients of Variation (CV) (Fig. S5c). Regional averaged TRMM P was higher than the observed average (Fig. S5d). This is expected because TRMM can observe heavy precipitation over high altitudes while most stations are located at lower altitude areas with less precipitation[12,13,14].

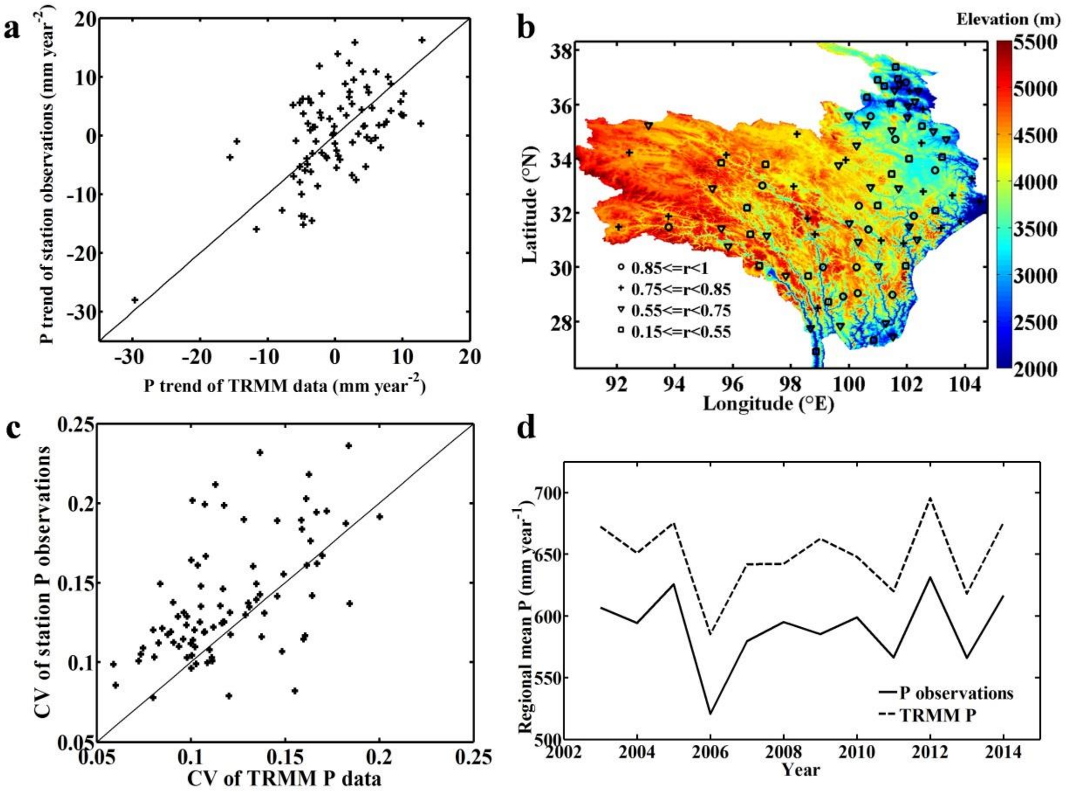

Figure S5. Evaluation of TRMM *P* data by comparing against *P* observations at 87 meteorological stations over 2003–2014. (a) Scatterplot of *P* trends. (b) Distribution of correlation coefficients. Elevation and station locations are shown, with stations denoted with different symbols according to the correlation coefficients. **(c) Scatterplot of Coefficients of Variation (CV).** CV is calculated as $std(P)/mean(P)$. **(d) Comparison of regional mean values.** These figures were generated

using MATLAB R2012b (v 8.0.0.783, http://www.mathworks.com) © The MathWorks.

Second, we checked the performance of TRMM data in constructing the inter-annual variation in *Q*. Other precipitation dataset, including west01[15], MSWEP[16], nearest interpolation results of the monthly gauge observed *P*, and thin-plate regression interpolation[17] results using TRMM as a covariate, were also considered for comparison. Results show that the inter-annual variation in *Q* at five gauges can be best explained with the raw TRMM *P* data (Table S4). Specifically, TRMM *P* provided the best validation statistics for Luning, Zhimenda and Jiayuqiao basins, and achieved acceptable accuracy for Tangnaihai and Changdu basins. In comparison, other *P* dataset produced problematic results for Zhimenda basin and/or Jiayuqiao basin, though the results for Tangnaihai and Changdu basins were slightly better. It is noted that in our study we only focused on inter-annual *P* data variations, which is insensitive to systematic errors. If the aim is to estimate the absolute values of *Q*, calibrations of *P* should be considered.

Table S4. Correlation coefficient between observed and estimated annual *Q* time-series using different precipitation datasets. The *P* dataset producing the highest value is bolded for each row.

Gauge names	Raw TRMM	MSWEP	west01	Nearest interpolation	Thin plate regression spline interpolation using raw TRMM as a covariate
Tangnaihai	0.77	**0.91**	0.76	0.86	0.79
Changdu	0.78	0.67	**0.84**	0.82	0.79
Zhimenda	**0.73**	0.14	0.14	0.28	0.46
Jiayuqiao	**0.76**	0.30	0.67	0.69	0.69
Luning	**0.80**	0.75	0.71	0.77	0.79

References

1 Hijmans, R. J., Cameron, S. E., Parra, J. L., Jones, P. G. & Jarvis, A. Very high resolution interpolated climate surfaces for global land areas, *International Journal of Climatology* **25**, 1965–1978, doi:10.1002/joc.1276 (2005).

2 Leuning, R., Zhang, Y. Q., Rajaud, A., Cleugh, H., & Tu, K. A simple surface conductance model to estimate regional evaporation using MODIS leaf area index and the Penman-Monteith equation. *Water Resources Research* **44**, W10419, doi:10.1029/2007WR006562 (2008).

3 Zhang, Y. Q. *et al*. Multi-decadal trends in global terrestrial evapotranspiration and its components. *Scientific Report* **6**, 19124; doi: 10.1038/srep19124 (2016).

4 Miralles, D. G. *et al*. El Niño–La Niña cycle and recent trends in continental evaporation. *Nature Climate Change* **4**, 122–126, DOI: 10.1038/NCLIMATE2068 (2014).

5 Mu, Q., Zhao, M. & Running S. W. Improvements to a MODIS Global Terrestrial Evapotranspiration Algorithm. *Remote Sensing of Environment* **115**, 1781–1800, doi:10.1016/j.rse.2011.02.019 (2011).

6 Zhang, K., Kimball, J. S., Nemani, R. & Running, S. W. A continuous satellite-derived global record of land surface evapotranspiration from 1983 to 2006. *Water Resources Research* **46**, W09522 (2010).

7 Reager, J. T. & Famiglietti, J. S. Characteristic mega-basin water storage behaviour using GRACE. *Water Resources Research* **49**, 3314–3329 (2013).

8 Bi, Y. B., Xie, H. J., Huang, C. L. & Ke, C. Q. Snow Cover Variations and Controlling Factors at Upper Heihe River Basin, Northwestern China. *Remote Sensing* **7**, 6741-6762; doi:10.3390/rs70606741 (2015).

9 Pieczonka, T. & Bolch, T. Region-wide glacier mass budgets and area changes for the Central Tien Shan between 1975 and 1999 using Hexagon KH-9 imagery. *Global and Planetary Change* **128**, 1–13 (2015).

10 Watkins, M. M., Wiese, D. N., Yuan, D. N., Boening, C. & Landerer, F. W. Improved methods for observing Earth’s time variable mass distribution with

GRACE using spherical cap mascons. *Journal of Geophysical Research Solid Earth* **120**, 2648–2671, doi:10.1002/2014JB011547 (2015).

11 Wiese, D. N., Landerer, F. W. & Watkins, M. M. Quantifying and reducing leakage errors in the JPL RL05M GRACE mascon solution, *Water Resources Research* **52**, 7490–7502, doi:10.1002/2016WR019344 (2016).

12 Immerzeel, W. W., Wanders, N., Lutz, A. F., Shea, J. M. & Bierkens, M. F. P. Reconciling high-altitude precipitation in the upper Indus basin with glacier mass balances and runoff, *Hydrology and Earth System Sciences* **19** (11), 4673–4687 (2015).

13 Mishra, V. Climatic uncertainty in Himalayan Water Towers, *Journal of Geophysical Research Atmospheres* **120**, 2689–2705, doi:10.1002/2014JD022650 (2015).

14 Palazzi, E., von Hardenberg, J. & Provenzale, A. Precipitation in the Hindu-Kush Karakoram Himalaya: Observations and future scenarios, *Journal of Geophysical Research Atmospheres* **118**, 85–100, doi:10.1029/2012JD018697(2013).

15 He, J. & Yang, K. China Meteorological Forcing Dataset. *Cold and Arid Regions Science Data Center at Lanzhou*, doi:10.3972/westdc.002.2014.db (2011).

16 Beck, H. E. *et al*. MSWEP: 3-hourly 0.25 ° global gridded precipitation (1979–2015) by merging gauge, satellite, and reanalysis data. *Hydrology and Earth System Sciences* **21**(1), 589-615 (2017).

17 Wood, S. N. *Generalized Additive Models: An Introduction With R*; Chapman & Hall/CRC: Boca Raton, FL, USA, (2006).
